# Advance directives among community-dwelling stroke survivors

**DOI:** 10.1371/journal.pone.0292484

**Published:** 2023-10-17

**Authors:** Soumya Gupta, Bridget J. Chen, Deji Suolang, Rachel Cooper, Roland Faigle

**Affiliations:** Department of Neurology, Johns Hopkins University School of Medicine, Baltimore, Maryland, United States of America; Epicentre, FRANCE

## Abstract

**Objective:**

Advance directives (ADs) are integral to health care, allowing patients to specify surrogate decision-makers and treatment preferences in case of loss of capacity. The present study sought to identify determinants of ADs among stroke survivors.

**Methods:**

In this cross-sectional study (Care Attitudes and Preferences in Stroke Survivors [CAPriSS]), community-dwelling stroke survivors were surveyed on ADs; validated scales were used to query palliative care knowledge and attitudes towards life-sustaining treatments. Logistic regression was used to determine variables associated with ADs.

**Results:**

Among 562 community-dwelling stroke survivors who entered the survey after screening questions confirmed eligibility, 421 (74.9%) completed survey components with relevant variables of interest. The median age was 69 years (IQR 58–75 years); 53.7% were male; and 15.0% were Black. Two hundred and fifty-one (59.6%) respondents had ADs. Compared to stroke survivors without ADs, those with ADs were more likely to be older (median age 72 vs. 61 years; p<0.001), White (91.2% vs. 75.9%, p<0.001), and male (58.6% vs. 46.5%, p = 0.015), and reported higher education (p<0.001) and income (p = 0.011). Ninety-eight (23.3%) participants had “never heard of palliative care”. Compared to participants without ADs, participants with ADs had higher Palliative Care Knowledge Scale (PaCKS) scores (median 10 [IQR 5–12] vs. 7 [IQR 0–11], p<0.001), and lower scores on the Attitudes Towards Life-Sustaining Treatments Scale (indicating a more negative attitude towards life-sustaining treatments; median 23 [IQR 18–28] vs. 29 [IQR 24–35], p<0.001). Multivariable logistic regression identified age (OR 1.62 per 10 year increase, 95% CI 1.30–2.02; p<0.001), prior advance care planning discussion with a physician (OR 1.73, 95% CI 1.04–2.86; p = 0.034), PaCKS scores (OR 1.06 per 1 point increase, 95% CI 1.01–1.12; p = 0.018), and Attitudes Towards Life-Sustaining Treatments Scale scores (OR 0.91 per 1 point increase, 95% CI 0.88–0.95; p<0.001) as variables independently associated with ADs.

**Conclusions:**

Age, prior advance care planning discussion with a physician, palliative care knowledge, and attitudes towards life-sustaining treatments were independently associated with ADs.

## Introduction

Stroke is a principal cause of mortality and long-term disability [1, 2], and survivors of stroke are at an increased risk of recurrent stroke, post-stroke disability, and premature death [3–6]. All patients, but particularly those who are at high risk for future severe illness, are encouraged to engage in advance care planning (ACP) and have advance directives (ADs). Professional societies recognize discussions regarding goals of care and documentation of advance directives as key measures of quality care in patients with neurological illnesses [7, 8]. ACP, i.e. the process by which care preferences in the face of future illness are discussed and communicated, and ADs, i.e. a legal document that designates a surrogate decision-maker and specifies preferences of care in the context of loss of healthcare decision-making capacity, are quality indicators of care [8], yet, are underutilized after stroke [9–12]. ADs prevent unwanted hospitalizations and futile treatments, promote patient-centered and goal-concordant care, and reduce decisional burden, stress, and anxiety among family members [13–16]. The determinants of ADs in stroke patients are poorly understood. In addition to specific demographic characteristics, AD completion may depend on patients’ prior ACP discussions with their physician(s) [10], but may also depend on perceptions, attitudes, preferences, and knowledge surrounding palliative care and life-sustaining treatments.

Effective interventions to increase AD use in stroke survivors are scarce. In the present study, we aimed to identify determinants of ADs among community-dwelling stroke survivors. Specifically, we hypothesized that palliative care knowledge and general preferences regarding aggressiveness of care are drivers of AD completion. The identification of determinants of ADs after stroke might enable interventions specifically addressing the drivers of AD, thus resulting in strategies to effectively increase AD use. Similarly, clinicians’ awareness of potential drivers of ADs may facilitate patient-centered discussions surrounding ACP, and may thereby promote the use of ADs among stroke survivors.

## Materials and methods

### Study population

Deidentified data that support the findings of this study are available at xxx. In the cross-sectional Care Attitudes and Preferences in Stroke Survivors (CAPriSS) study, community-dwelling stroke survivors residing in Maryland were surveyed. Adult Black or White participants were eligible to participate if they had a self-reported history of an ischemic or hemorrhagic stroke in the last 10 years and were living in Maryland. Institutionalized or non-English speaking individuals were excluded. Potential participants were identified from the electronic medical records (EMR) by the Johns Hopkins Core for Clinical Research Data Acquisition (CCDA). CCDA identified individuals fulfilling inclusion and exclusion criteria with at least one in- or outpatient visit within the Johns Hopkins Health System in Maryland between January 1, 2016, and December 31, 2019. Only patients with a stroke diagnosis documented in the EMR were included, regardless of whether the stroke was the reason for the visit or whether the stroke diagnosis was made within the Johns Hopkins Health System. Due to potential diagnostic misclassifications in the EMR, all survey participants answered screening questions to confirm that inclusion and exclusion criteria were fulfilled. The diagnosis of stroke was ascertained by asking potential participants “Has a doctor ever told you that you have had a stroke?”. Potential participants who were (incorrectly) identified as having had a stroke based on the EMR but denied a stroke diagnosis during the screening questions “screened out” and were not able to enter the survey. The study protocol was approved by the Johns Hopkins University School of Medicine institutional review board.

### Survey proceedings

Eligible participants were invited to participate in an online survey on perceptions and attitudes regarding stroke. Potential participants were contacted by e-mail (if available) or by mailed postcard containing basic information on the nature of the study and the URL to the online survey. The survey was prepared on the Johns Hopkins Qualtrics platform. Survey responses were collected between June 11, 2021 and September 10, 2021. Participants who did not complete the survey received up to 2 reminder e-mails or postcards throughout the study period. Participants who completed the study received a $5 Amazon gift card to compensate for their time.

### Survey design

After the completion of screening questions to ensure eligibility, participants answered questions on demographics, socioeconomic information, comorbidities, and their stroke diagnosis, followed by questions regarding attitudes, preferences, knowledge, and perceptions regarding illness severity, end-of-life care, and life-prolonging interventions. Answer choices were either multiple choice or presented on a Likert scale. Only responses from participants with complete response for variables of interest were included.

Embedded in the survey were validated scales to assess palliative care knowledge and general attitudes towards life-sustaining treatments. Specifically, participants were asked “How would you describe your level of knowledge about palliative care?”, followed by the answer choices “I have never heard of it”, “I know a little bit about palliative care”, and “I know what palliative care is and could explain it to somebody else”, as previously described [17, 18]. Participants who indicated some level of palliative care knowledge completed the Palliative Care Knowledge Scale (PaCKS), a validated 13-item scale (range of scores from 0 to 13; higher scores indicate higher level of knowledge) [19–22]. Participants who indicated that they had “never heard of palliative care” were assigned a PaCKS score 0.

In order to assess preferences for aggressiveness of care, participants completed the General Attitudes Towards Life-sustaining Treatments Scale, a validated 13-item survey instrument to assess attitudes towards life-sustaining treatments [23, 24]. Negatively worded questions were coded in the reverse. The total scale scores range from 13 to 52; higher scores indicating a greater preference for aggressive treatment.

### Statistical analysis

Survey responses among patients with and without ADs were compared using Chi-square and Wilcoxon rank-sum tests for categorical and continuous variables, respectively. Cronbach’s alpha was calculated for the PaCKS and the Attitudes Towards Life-sustaining Treatment Scale to assess internal consistency. Multivariable logistic regression was used to identify variables independently associated with ADs. Model covariates included variables associated with ADs in univariate analysis as well as other potentially relevant variables. Receiver operating characteristics analysis was conducted for a model including only variables independently associated with ADs. Likelihood ratio testing was performed to compare model fit between the model containing only variables independently associated with the ADs and the complete model containing all covariates. Statistical analyses were conducted using Stata version 15 (College Station, TX). Statistically significant results were defined as *p*<0.05, and 95% confidence intervals are reported.

## Results

### Study population characteristics and advance directives

**Fig 1:**
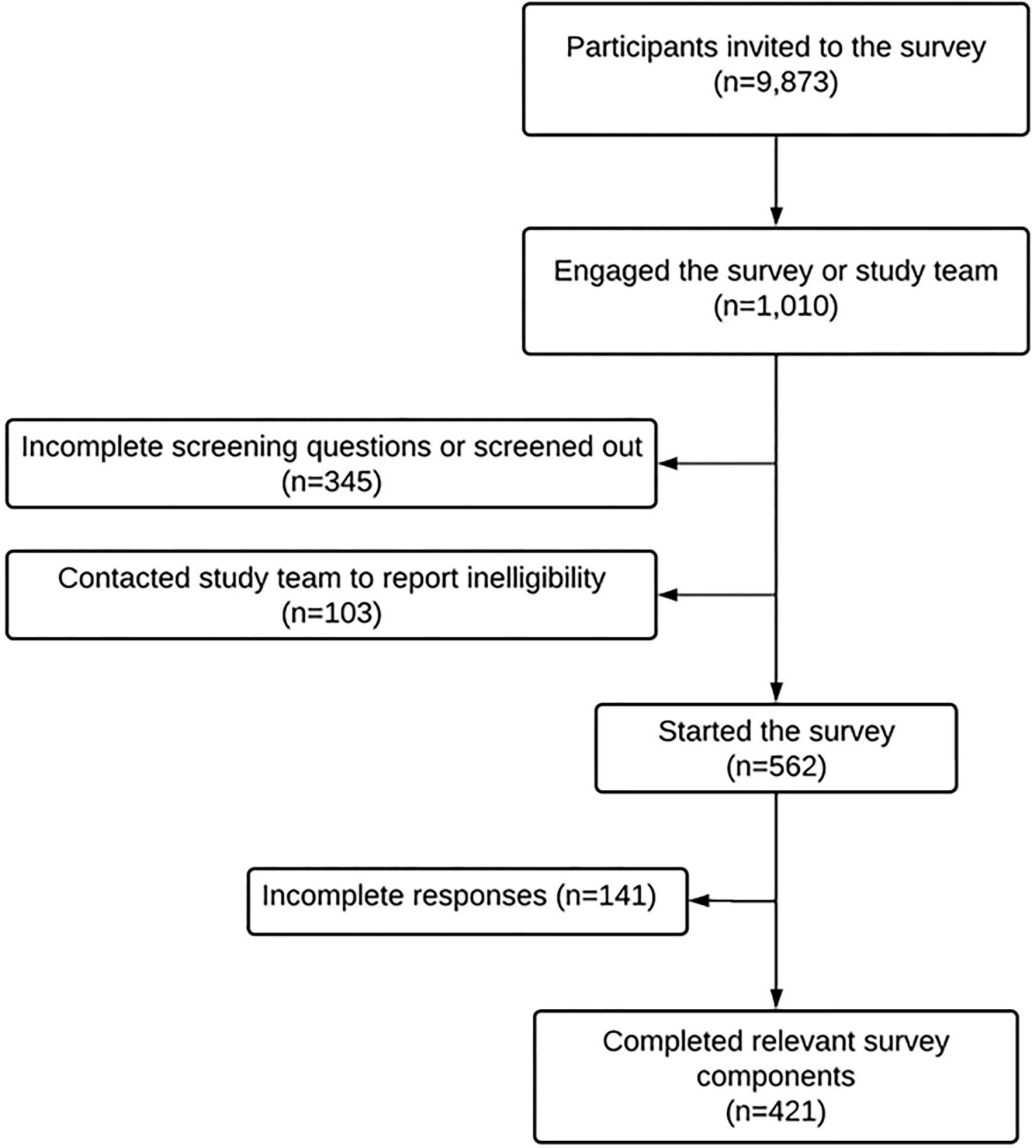
Flow diagram indicating the derivation of the study sample.

Fig 1 illustrates the derivation of the analytic sample. A total of 1,010 (10.2%) of those who were invited to participate engaged the survey. Among the 562 community-dwelling stroke survivors who entered the survey after screening questions confirmed eligibility, 421 completed survey components with relevant variables of interest. The median age was 69 years (IQR 58–75 years); 53.7% were male; and 15.0% were Black (Table 1). Two hundred and fifty-one (59.6%) respondents indicated that they have ADs. Compared to stroke survivors without ADs, those with ADs were older (median age 72 vs. 61 years; p<0.001) and were more likely to be White (91.2% vs. 75.9%, p<0.001), male (58.6% vs. 46.5%, p = 0.015), higher educated (p<0.001), and report higher annual income (p = 0.011; Table 1). Respondents with ADs were more likely to have had prior discussions about advance care planning with their physicians that those without ADs (68.5% vs. 58.8%, p = 0.041). Table 1 shows the baseline characteristics of patients with and without ADs.

**Table 1:** Baseline characteristics of the study population.

Characteristics	All *(n = 421)*	No Advance Directives *(n = 170)*	Advance Directives *(n = 251)*	p
Age–years: median (IQR)	69 (58–75)	62 (50–71)	72 (64–77)	<0.001
Race–n (%)				<0.001
White	358 (85.0)	129 (75.9)	229 (91.2)	
Black/African American	63 (15.0)	41 (24.1)	22 (8.8)	
Gender–male: n (%)	226 (53.7)	79 (46.5)	147 (58.6)	0.015
Education–n (%)				<0.001
Some high school or less	7 (1.7)	3 (1.8)	4 (1.6)	
High school diploma/equivalent	37 (8.8)	22 (12.9)	15 (6.0)	
Some college/university	92 (21.9)	45 (26.5)	47 (18.7)	
College/university degree (BA/BS)	118 (28.0)	57 (31.8)	64 (25.5)	
Graduate or professional degree	167 (39.7)	46 (27.1)	121 (48.2)	
Income (per year)–n (%)				0.011
< $20,000	25 (5.9)	14 (8.2)	11 (4.4)	
$20,000 - $49,999	47 (11.2)	27 (15.9)	20 (8.0)	
$50,000 - $74,999	45 (10.7)	15 (8.8)	30 (12.0)	
$75,000 - $99,999	57 (13.5)	24 (14.1)	33 (13.1)	
$100,000 - $149,999	85 (20.2)	29 (17.1)	56 (22.3)	
$150,000 - $250,000	67 (15.9)	25 (14.7)	42 (16.7)	
> $250,000	31 (7.4)	10 (5.9)	21 (8.4)	
Prefer not to say	64 (15.2)	26 (15.3)	38 (15.1)	
Religious importance–n (%)				0.660
Not at all/slightly	138 (32.8)	60 (35.3)	78 (31.1)	
Moderately	104 (24.7)	40 (23.5)	64 (25.5)	
Very/extremely	179 (42.5)	70 (41.2)	109 (43.4)	
Married–n (%)	279 (66.3)	110 (64.7)	169 (67.3)	0.576
Living alone–n (%)	92 (21.9)	36 (21.2)	56 (22.3)	0.782
Residual stroke symptoms–n (%)	260 (61.8)	109 (64.1)	151 (60.2)	0.412
mRS–n (%)				0.677
0–1	222 (52.7)	92 (54.1)	130 (51.8)	
2	142 (33.7)	58 (34.1)	84 (33.5)	
≥3	57 (13.5)	20 (11.8)	37 (14.7)	
Worry about another stroke–n (%)				0.253
Not at all/rarely	231 (54.9)	85 (50.0)	146 (58.2)	
Sometimes	144 (34.2)	64 (37.6)	80 (31.9)	
Frequently/all the time	46 (10.9)	21 (12.4)	25 (10.0)	
Previous ACP discussion with physician–n (%)	272 (64.6)	100 (58.8)	172 (68.5)	0.041
PaCKS score–median (IQR)	9 (0–12)	7 (0–11)	10 (5–12)	<0.001
Attitudes Towards Life-Sustaining Treatments Scale score–median (IQR)	25 (20–31)	29 (24–35)	23 (18–28)	<0.001

ACP: Advance care planning; mRS: modified Rankin Scale; PaCKS: Palliative Care Knowledge Scale.

### Palliative care knowledge and advance directives

**Fig 2:**
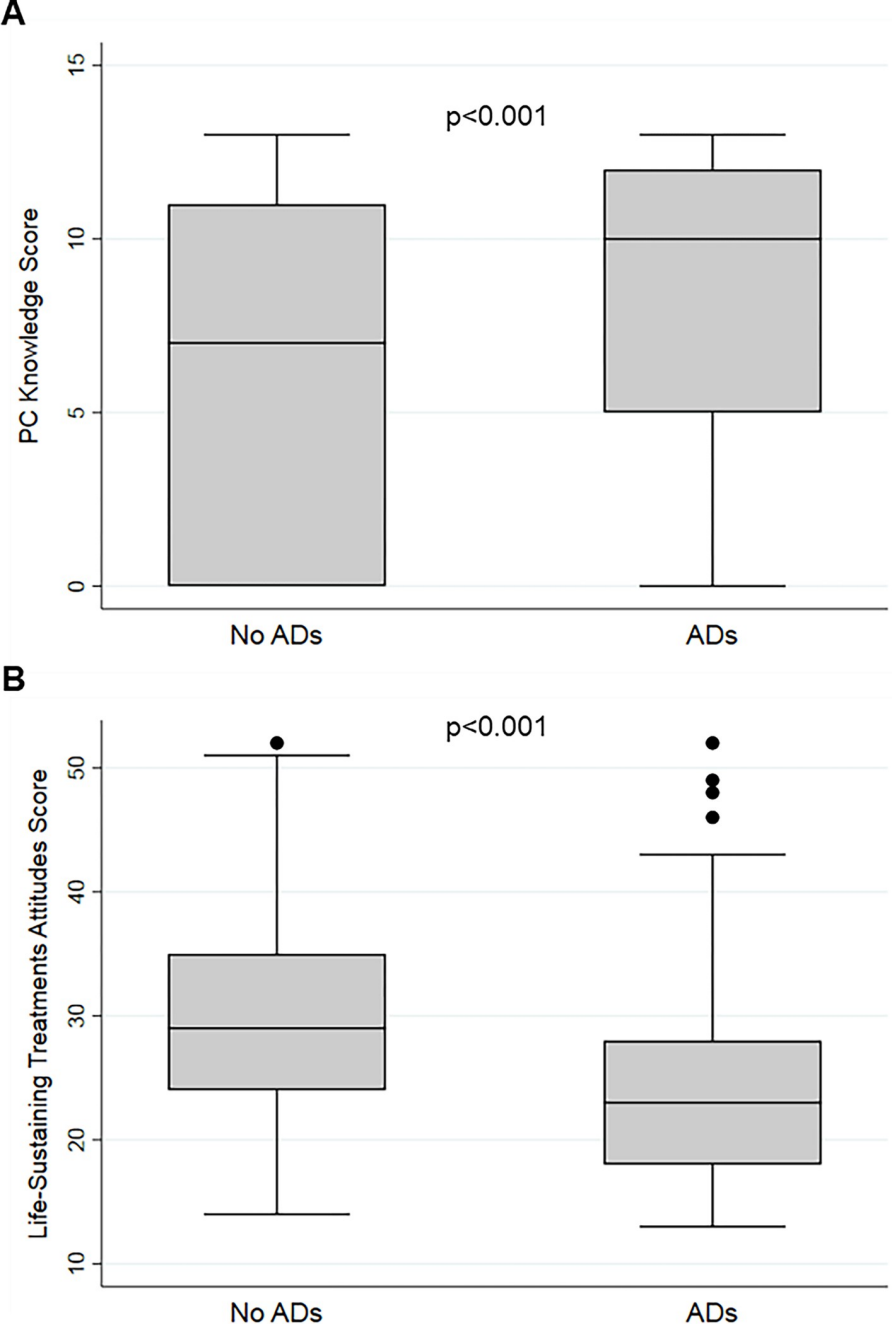
Box-and-Whisker plot of palliative care knowledge and attitudes towards life-sustaining procedures scores by AD status. (A) PC knowledge. (B) Attitudes towards life-sustaining procedures. ADs: Advance directives. PC: palliative care.

Ninety-eight (23.3%) participants responded that they had “never heard of palliative care”. The median score on the 13-item Palliative Care Knowledge Scale (PaCKS; range of scores 0–13) was 9 (IQR 0–12; Table 1); 65.6% of respondents had a PaCKS score ≥6. Cronbach’s alpha of the PaCKS was 0.890, indicating excellent internal consistency among the items of the scale in the study sample. Response patterns to the individual items of the PaCKS are presented in S1 Table. Participants with ADs had significantly higher PaCKS scores than those without ADs (median 10 [IQR 5–12] vs. 7 [IQR 0–11], p<0.001; Fig 2A). In unadjusted logistic regression, every 1 point increase in PaCKS score was associated with an 8% increase in odds of having ADs (OR 1.08, 95% CI 1.04–1.12; Table 2). Participants with a PaCKS score ≥6 were more likely to have ADs than those with a PaCKS score <6 (66.7% vs. 46.2%, p<0.001; unadjusted OR 2.33, 95% CI 1.54–3.51).

**Table 2:** Crude (unadjusted) and adjusted logistic regression models for variables associated with ADs.

Variable	Unadjusted OR (95% CI)	Adjusted OR (95% CI)
Age (per 10 years)	1.74 (1.48–2.06)	1.62 (1.30–2.02)
Prior ACP discussion with physician	1.52 (1.02–2.29)	1.73 (1.04–2.86)
PaCKS score (per 1 point)	1.08 (1.04–1.12)	1.06 (1.01–1.12)
Attitudes Towards Life-Sustaining Treatments Scale score (per 1 point)	0.90 (0.88–0.93)	0.91 (0.88–0.95)

ACP: Advance care planning; ADs: Advance directives; PaCKS: Palliative Care Knowledge Scale.

### Attitudes towards life-sustaining treatments and advance directives

The median score on the Attitudes Towards Life-Sustaining Treatments Scale (range of scores 13–52) was 25 (IQR 20–31; Table 1); Cronbach’s alpha was 0.867, indicating excellent internal consistency. Response patterns to the individual items of the Attitudes Towards Life-Sustaining Treatments Scale are presented in S2 Table. Participants with ADs had significantly lower scores (indicating a more negative attitude towards life-sustaining treatments) than those without ADs (median 23 [IQR 18–28] vs. 29 [IQR 24–35], p<0.001; Fig 2B). In unadjusted logistic regression, every 1 point increase in scores was associated with decreased odds of having ADs (OR 0.90, 95% CI 0.88–0.93; Table 2).

### Variables independently associated with advance directives

Multivariable logistic regression identified age (p<0.001; Table 2), prior advance care planning discussion with a physician (p = 0.034), PaCKS scores (p = 0.018), and the Attitudes Towards Life-Sustaining Treatments Scale scores (p<0.001) as variables independently associated with the presence of ADs (Table 2); gender, race, education, income, residual stroke symptoms, marital status, living alone, religious importance, mRS, and self-assessed stroke risk were not significantly associated with the presence of ADs. A model containing the variables independently associated with AD completion, i.e. age, prior advance care planning discussion with a physician, PaCKS scores, and the Attitudes Towards Life-Sustaining Treatments Scale scores achieved an area under the curve of 0.776 (95% confidence interval 0.732–0.820). Likelihood ratio (LR) testing showed that the addition of the remaining variables to the model did not improve the model fit (LR test p = 0.298).

## Discussion

In this study of community-dwelling stroke survivors, almost 60% of survey participants had documented ADs. Our study identified age, palliative care knowledge, negative attitudes towards life-sustaining treatments, and physician conversations regarding ACP as variables independently associated with ADs. AD completion rates were almost twice as high as in the general US population [25], consistent with previously reported AD rates in other neurological patient populations [25, 26]. AD rates are higher among stroke survivors than in the general population because 1) the average age of stroke survivors is higher than that of the general population (and ADs are associated with age); 2) the experience of a prior stroke may heighten a patient’s awareness surrounding health care choices and their implications; and 3) increased contact with the medical system and health care providers may prompt AD completion.

While age has previously been reported to be associated with AD in stroke and non-stroke patient populations [10, 27], our study also found an independent association between palliative care knowledge and ADs in stroke patients. More than two-thirds of participants indicated familiarity with palliative care, which is substantially higher than the US national average; for example, a study analyzing data from the Health Information National Trends Survey reported that just over 30% of participants had knowledge of palliative care [17, 18]. Although the PaCKS has not previously been administered in a nationally representative sample, the median PaCKS score of 9 in our sample was considerably higher than in a prior study [21] of laypersons that reported a median PaCKS score of 5; higher participant age, income, and level of education among the stroke survivors in our study may partially explain this discrepancy. In addition, a focus on palliative care in medical education and training in recent years may have contributed to an increase in familiarity with palliative care particularly among the younger generation of stroke providers. Correspondingly, stroke survivors may have been exposed to palliative care during their index stroke event and the associated acute care, or during subsequent outpatient care and follow-up visits. The association between palliative care knowledge and ADs in our study is consistent with a prior study describing an increase in rates of ADs amongst patients with neurological illnesses after receiving palliative care consultations [26].

Prior ACP discussions with a physician as a driver of ADs are consistent with prior studies in stroke and non-stroke patient populations [10, 16]. These data underline the importance and effectiveness of ACP discussions regarding AD completion, and highlight the need to standardize ACP into care processes during outpatient visits. Our data lend credence to the notion that information-giving and education about palliative care in the context of patient-provider ACP discussions may increase AD completion rates among stroke patients. Efforts to increase palliative care knowledge in the population in general, and among stroke survivors specifically, may thus be a potential tool to increase ACP awareness and AD use.

In our study, participants with a positive attitude towards life-sustaining treatments were less likely to have ADs. This is in line with a study in cancer patients and their surrogates, in which patients who preferred more aggressive care had lower rates of ADs [28]. Although we were unable to explore the exact reasons for this association, patients with a preference for aggressive care may perceive ADs as unnecessary (since aggressive care is generally the default in the absence of ADs), or they may believe that the presence of ADs could result in care incongruent with their preference for aggressive care. For example, some patients may believe that the mere presence of an AD may result in inferior care or that physicians might not be fully invested in their care, especially among patients with low levels of trust in the healthcare system. Regardless of the underlying reasons, these data emphasize that patients with favorable views of life-sustaining interventions are at increased risk of not having ADs. Those patients may require additional counselling on the nature of ADs in order to clarify the purpose and utility of ADs, and providers may educate those patients that ADs may be beneficial to them even when aggressive care is preferred.

Our findings are consistent with prior studies in neurological and non-neurological patient populations describing lower rates of ADs in racial and ethnic minorities [10, 29–32]; however, while race was associated with ADs in univariate analysis in our study, race was not independently associated with ADs after accounting for palliative care knowledge and general attitudes towards life-sustaining treatments. Similarly, income and education were associated with ADs in univariate but not multivariable regression in a model that included PaCKS and attitude towards life-sustaining treatments. The attenuation of the direct effect of race, income, and education on ADs may lie in association of these variables with palliative care knowledge and attitude towards life-sustaining treatments. For example, income and education are associated with palliative care knowledge, and race is associated with attitude towards life-sustaining treatments. Palliative care knowledge and attitude towards life-sustaining treatments may thus partially mediate the effects of race, income, and education on ADs, and in a model testing the association between palliative care knowledge and attitudes towards life-sustaining treatments with ADs, other variables such as race, income, and education lose statistical significance.

Strengths of this study in stroke survivors include the relatively large sample size, and the granular evaluation of palliative care knowledge and attitudes towards life-sustaining treatments with validated scales. Limitations include the cross-sectional design. Residual stroke symptoms interfering with the completion of this survey may have prevented some community dwelling stroke survivors from participating. In addition, our study was limited to Black and White participants, and the results may not be generalizable to other racial or ethnic groups. The exclusion of non-English speakers limits generalization to this patient population and may have contributed to the relatively high rate of ADs in our study. Our survey response rate was 10.2%. Although this response rate is at the lower end of survey response rates, this rate is a reflection of respondents among those who were contacted but not among those who were eligible. Our algorithm to identify potential participants was intentionally overly sensitive (in order to not miss any potential stroke survivor) but lacked specificity; thus, the need for screening questions. Therefore, it is likely that there were numerous other participants who became aware of the survey and considered participating, but realized that they were ineligible based on the information provided even before starting the screening questions. For example, several participants did not formally engage the online survey but e-mailed the study team to report that they were ineligible because they did not have a stroke. Consequently, it is likely that numerous other similarly ineligible potential participants merely did not take the step to inform the study team of their ineligibility. Potential participants were contacted with e-mail and residential information that were up to several years old and thus potentially outdated in some instances. Therefore, some of the potential participants we intended to contact were likely not reached, or were deceased or institutionalized at the time of the survey. As such, it is likely that our measured response rate significantly underestimates the true response rates among eligible participants. Lastly, pre-coded response options may not have fully captured the nuanced attitudes and perceptions of all respondents; we tried to mitigate this by using established scales that have previously been validated in various settings. Despite these limitations, our results identified modifiable factors that may aid in increasing AD rates among stroke survivors. Our data highlight the importance of ongoing and recurrent ACP discussions and the need for palliative care education of patients and family members as a part of routine in- and outpatient stroke care to increase rates of AD completion [26, 33].

## Supporting information

S1 Checklist STROBE (Strengthening The Reporting of OBservational Studies in Epidemiology) checklist. (PDF)

S1 Table Response patterns to the individual Palliative Care Knowledge Scale items in % (n = 421). *Indicates the correct response.
(DOCX)

S2 Table Response patterns to the items of the general attitudes towards life-sustaining treatments scale in % (n = 421). (DOCX)
