# Supplementary material for: Advance directives among community-dwelling stroke survivors
Source: PLoS One. 2023 Oct 17;18(10):e0292484. doi: 10.1371/journal.pone.0292484 (PMC10581473; doi:10.1371/journal.pone.0292484)
Supplement: S1 Table — *Indicates the correct response. (DOCX) [file pone.0292484.s002.docx]

# SUPPLEMENTAL INFORMATION

**Advance Directives Among Community-Dwelling Stroke Survivors**

**S1 Table. Response patterns to the individual Palliative Care Knowledge Scale items in % (n=421).** *Indicates the correct response.

| Item | True | False | Don’t  Know |
| --- | --- | --- | --- |
| A goal of palliative care is to address any psychological issues brought up by  serious illness | 53.7* | 10.5 | 35.9 |
| Stress from serious illness can be  addressed by palliative care | 57.5* | 7.1 | 35.4 |
| Palliative care can help people manage the  side effects of their medical treatments | 59.4* | 6.7 | 34.0 |
| When people receive palliative care, they  must give up their other doctors | 6.7 | 54.2* | 39.2 |
| Palliative care is exclusively for people  who are in the last six months of life | 19.7 | 37.8* | 42.5 |
| Palliative care is specifically for people  with cancer | 2.4 | 63.0* | 34.7 |
| People must be in the hospital to receive  palliative care | 1.4 | 59.6* | 39.0 |
| Palliative care is designed specifically for  older adults | 4.5 | 60.6* | 34.9 |
| Palliative care is a team-based approach to  care | 58.0* | 4.8 | 37.3 |
| A goal of palliative care is to help people  better understand their treatment options | 51.5* | 12.8 | 35.6 |
| Palliative care encourages people to stop  treatments aimed at curing their illness | 9.7 | 50.4* | 39.9 |
| A goal of palliative care is to improve a person’s ability to participate in daily  activities | 46.8* | 17.8 | 35.4 |
| Palliative care helps the whole family  cope with a serious illness | 64.4* | 3.3 | 32.3 |
