# Supplementary material for: Advance directives among community-dwelling stroke survivors
Source: PLoS One. 2023 Oct 17;18(10):e0292484. doi: 10.1371/journal.pone.0292484 (PMC10581473; doi:10.1371/journal.pone.0292484)
Supplement: S2 Table — (DOCX) [file pone.0292484.s003.docx]

# SUPPLEMENTAL INFORMATION

**S2 Table. Response patterns to the items of the General Attitudes Towards Life-Sustaining Treatments Scale in % (n=421).**

| Item | Disagree | Somewhat  Disagree | Somewhat  Agree | Agree |
| --- | --- | --- | --- | --- |
| If life-prolonging technology exists, it should always  be used | 23.5 | 18.1 | 37.3 | 21.1 |
| Doctors should generally try to keep their patients alive on machines for as long as possible, no matter  how uncomfortable the machines are | 59.4 | 23.3 | 13.1 | 4.3 |
| If a patient is dying, it is best not to prolong their  lives by medical means | 13.3 | 21.4 | 33.3 | 32.1 |
| Life sustaining machines should never be stopped  even if the patient appears to be dying because there is always the chance of a miracle | 50.4 | 30.4 | 12.6 | 6.7 |
| It is a doctor's duty to stop life prolonging treatments of patients if the patient does not want them  anymore | 7.6 | 5.0 | 20.4 | 67.0 |
| Even if I were terminally ill, I would want  everything done to keep me alive as long as possible | 45.1 | 25.2 | 19.7 | 10.0 |
| Life sustaining machines are often painful | 14.5 | 27.3 | 42.5 | 15.7 |
| When a person is permanently unconscious (in a coma), with no hope of waking up, medical treatments usually should not be used to keep them  alive | 6.9 | 15.2 | 28.7 | 49.2 |
| Even if my condition is hopeless I would want my  life prolonged as much as possible | 56.8 | 20.9 | 14.5 | 7.8 |
| I would not want machines used to keep me alive | 16.2 | 19.2 | 25.4 | 39.2 |
| Present day medical care frequently prolongs dying  without providing any real benefit to the patient | 11.4 | 24.0 | 32.5 | 30.0 |
| If a patient is unable to breathe without a breathing machine, it would be wrong to take them off the machine (even if the condition is hopeless) because  that would be killing the patient | 48.0 | 26.1 | 16.2 | 9.7 |
| The use of life sustaining machines can be  humiliating to the patient | 22.8 | 22.8 | 31.8 | 22.6 |
